# Association of *Klotho* Gene Polymorphism and Serum Level of α Klotho Protein with Different Tumor Grades, Overall Survival and Cytokine Profile in Glioma Patients

**DOI:** 10.3390/ijms26010330

**Published:** 2025-01-02

**Authors:** Eszter Zsemlye, Vladimira Durmanova, Kristina Kluckova, Jan Kozak, Boris Rychly, Marian Svajdler, Viktor Matejcik, Monika Homolova, Juraj Steno, Luba Hunakova, Maria Bucova

**Affiliations:** 1 Institute of Immunology, Faculty of Medicine, Comenius University Bratislava, 813 72 Bratislava, Slovakia; eszter.zsemlye@fmed.uniba.sk (E.Z.); kristina.kluckova@fnnitra.sk (K.K.); monika.homolova@fmed.uniba.sk (M.H.); luba.hunakova@fmed.uniba.sk (L.H.); maria.bucova@fmed.uniba.sk (M.B.); 2 Clinic for Children and Adolescents, Faculty Hospital Nitra, 950 01 Nitra, Slovakia; 3 Department of Neurosurgery, Faculty of Medicine, Comenius University and University Hospital, 833 05 Bratislava, Slovakia; kozakjan@hotmail.com (J.K.); viktor.matejcik@fmed.uniba.sk (V.M.); juraj.steno@fmed.uniba.sk (J.S.); 4 Unilabs Ltd., 841 01 Bratislava, Slovakia; rychly.boris@alphamedical.sk; 5 Cytopathos Ltd., 831 03 Bratislava, Slovakia; svajdler@yahoo.com; 6 Sikl’s Department of Pathology, Faculty of Medicine and Faculty Hospital in Pilsen, Charles University, 306 05 Pilsen, Czech Republic

**Keywords:** cytokines, glioma, inflammation, *Klotho* gene, α klotho, overall survival

## Abstract

Gliomas are the most common and lethal forms of malignant brain tumors. We attempted to identify the role of the aging-suppressor *Klotho* gene and Klotho protein in the immunopathogenesis of gliomas. We examined *Klotho* genetic variants by PCR-RFLP and measured serum Klotho levels using the ELISA method. We found a statistically significantly increased frequency of rs1207568A allele and rs1207568 GA genotypes in co-dominant, dominant and over-dominant models in grade IV as compared to grade II and III glioma patients. The levels of soluble α Klotho (sαKL) were significantly lower in grade III and IV glioma patients than in healthy controls (*p* = 0.034; 0.0083). Patients with sαKL levels above 2500 pg/mL survived significantly longer than patients with sαKL below 2500 pg/mL (*p* = 0.038). We also found a positive correlation of the serum levels of sαKL with seven biomarkers, like angiogenic vascular endothelial growth factor (*p* = 0.0008), chemokine fractalkine (*p* = 0.0009), interferon γ (*p* = 0.003), glial derived neurotrophic factor (*p* = 0.0268), pro-inflammatory and pro-Th1 cytokine IL-6 (*p* = 0.0347), anti-inflammatory, pro-Th2 cytokines IL-4 (*p* = 0.0037) and IL-13 (*p* = 0.0004). Our results suggest the impact of *Klotho* genetic variants and Klotho levels on advanced-grade glioma.

## 1. Introduction

Gliomas belong to primary malignant tumors in the brain originating in glial cells and represent the most common form of central nervous system (CNS) neoplasms. Grade IV gliomas, termed glioblastomas (GBM), are the most frequent (70–75% of all glioma diagnoses), aggressive and lethal form of gliomas, with a median overall survival (OS) time of 14–17 months [1,2]. Despite improvements in the median- and short-term overall survival, the percentage of patients with glioblastoma achieving 5-year OS remains very low—4.6% [3]. The 10-year survival rate is estimated to be 0.71% [4]. The current standard of treatment includes maximal surgical resection and combined radio-chemotherapy [5].

According to the up-to-date WHO classification of CNS tumors, the heterogeneous group of gliomas in adults are grouped into diffuse gliomas and circumscribed (non-diffuse) astrocytic gliomas [6,7,8].

The aging-suppressor *Klotho* gene was identified by Kuro et al. in 1997 through a mutation of its promoter region in mice associated with a syndrome resembling aging [9]. Its name originates from Greek mythology, where Klotho (Clotho) spun the thread of life and controlled the ultimate destiny of humans [9]. An insertion mutation in the 5′ flanking region of the *αKlotho* gene in mice was associated with several symptoms of premature aging, such as atherosclerosis, osteoporosis, overall shorter life span, etc. [9,10].

In humans, this gene is located on chromosome 13 and consists of five exons and four introns in the coding region of *KL*. In the promoter region of the *KL* gene located in humans on chromosome 13, several consensus sequences were identified to bind to different transcription factors and thus be involved in multiple molecular signaling pathways [11]. This can explain the broad spectrum of its biological activities (anti-aging, healthspan- and lifespan-extending, cognitive-enhancing, anti-oxidative, anti-inflammatory, role in mineral metabolism, anti-tumor, etc.) [11].

The Klotho gene encodes three types of proteins: αKlotho proteins (αKL), β-Klotho proteins (βKL) and Klotho-related protein (Klrp; γ Klotho protein, also referred as *LCTL*) [12,13]. Moreover, three types of αKlotho proteins have been detected: the full-length transmembrane αKlotho (expressed primarily in the kidneys and in the choroid plexus of the brain), soluble αKlotho and secreted αKlotho [9]. Soluble αKlotho is produced due to cleavage of its membrane form by α- and β-secretases and by insulin stimulation. In contrast, secreted Klotho is generated due to alternative RNA splicing. Both soluble and secreted αKlotho are part of circulating αKlotho; however, the secreted αKlotho is the major form of circulating αKlotho. These circulating αKlotho may act as hormones and regulate functions in tissues or cells that do not express Klotho. This may partially explain why mutation of the gene *Klotho* causes extensive aging phenotypes despite being expressed in only a few tissues [11,14,15].

While membrane αKL has a function as a co-receptor of fibroblast growth factor (FGF), which regulates phosphate homeostasis and vitamin D metabolism [16,17,18], the secreted KLα is a regulator of oxidative stress activity, multiple growth factor receptors and ion channels [19,20]. Soluble Klotho in the circulation seems to be the primary mediator of anti-fibrotic effects. In humans, serum levels of αKlotho decrease with age after 40 years of life [21].

Overexpression of hormone αKL in mice extended their lifespan at a rate of 20–30% [22]. Other studies revealed deficits of memory retention, reduced number of synapses in the hippocampus, disturbed axonal transport, hippocampal degeneration and impaired myelin production in specific areas in *Klotho*-deficient mice [23]. Similar results were also obtained in humans [24,25].

Klotho is a well-established longevity hormone, and its most prominent function is the regulation of phosphate homeostasis. However, klotho also participates in multiple pleiotropic activities that are tightly associated with cancer and was discovered as a universal tumor suppressor [26,27,28]. Therefore, attention has been directed toward the association between cancer and αKL. The tumor suppressive sequence within the KL1 domain of the hormone Klotho was determined [29]. Doi et al. found that αKL inhibited TGF-β1 (transforming growth factor) signaling and suppressed cancer metastasis “in vivo” [30]. Moreover, Mota et al. pointed to the potential diagnostic and prognostic applications of Klotho in cancer [31].

Inflammation and the state of adaptive cell-mediated immunity are two main immunological factors influencing tumor growth [32]. In our study, we were interested whether the level of serum αKlotho affects the concentrations of the selected biomarkers of immunity and inflammation, as interferon γ (the main sustaining cytokine of the Th1 immunity with pro-inflammatory activity) and cytokines of the contra-regulatory Th2 immunity—IL-4 (with anti-inflammatory activity) and IL-13. We also analyzed the levels of pro-inflammatory cytokine IL-6, pro-inflammatory soluble TREM-1 (sTREM-1; triggering receptor expressed on myeloid cells-1) and sHLA-G (soluble human leukocyte antigen-G)—an immune checkpoint molecule with anti-inflammatory and immunosuppressive activities. We also focused on other biomarkers playing an important role in tumor formation and growth, such as angiogenic factor VEGF (vascular endothelial growth factor), GDNF (glial cell derived neurotrophic factor) and chemokine fractalkine/CX3CL1.

However, only limited data exist on Klotho’s role in gliomas. Peshes-Yeloz et al. found that αKlotho expression and methylation could predict the prognosis in patients with glioblastoma [33]. Jun Su and co-workers showed that high *LCTL* (*γ*Klotho) expression regulated by DNA methylation status was also significantly associated with high tumor aggressiveness and poor outcomes in glioma patients [34].

It is important to analyze whether there are some changes in the *Klotho* gene that precede the onset of the disease. *Klotho rs1207568* (c.-395G>A) is a single nucleotide variant (SNV) located in the promoter region of the *Klotho* gene, in which guanine nucleotide is replaced by adenine, whereas *Klotho rs564481* (c.1767C>T, p.His589) is a synonymous variant located in exon 4, where the cytosine nucleotide is substituted by thymine at codon 1767, resulting in no amino acid (histidine) change [35,36]. While Klotho’s role in tumorigenesis is well-established, its association with specific glioma-related genetic variants remains unexplored [37,38]. Therefore, our study aimed to investigate the association between Klotho gene polymorphisms (rs1207568 and rs564481) and overall survival in glioma patients. Additionally, we sought to elucidate the role of serum αKlotho levels in glioma pathogenesis and patient outcomes. Given the limited research on Klotho’s role in glioma and the absence of studies examining serum αKlotho levels in this context, our study aimed to fill these knowledge gaps.

## 2. Results

### 2.1. Characteristics of Glioma Patients and Selected Variables

Detailed parameters of the 55 enrolled patients with gliomas are summarized in Table 1. The average age at disease onset was 54.38 ± 15.22 years. According to histopathological characteristics, 15 patients were classified with grade II gliomas, 11 patients grade III and 29 patients grade IV gliomas. The time of overall survival (OS) ranged from 0 to 74 months, with an average survival of 50.47 ± 24.40 months for grade II, 32.45 ± 20.14 months for grade III and 9.57 ± 9.28 months for grade IV gliomas.

### 2.2. Analysis of Klotho rs1207568 and rs564481 Polymorphisms in Patients with Gliomas and Healthy Control Group

The allele and genotype frequencies of the *Klotho* rs1207568 (-395G/A) and rs564481 (1818C/T) gene polymorphisms observed in the glioma patients (n = 55) and healthy control subjects (controls; n = 140) are shown in Table 2. The genotype frequencies fit the Hardy–Weinberg equilibrium in the patients with gliomas (rs1207568: χ^2^ = 0.1148, *p* = 0.7347; rs564481: χ^2^ = 1.0911, *p* = 0.2962), as well as in the controls (rs1207568: χ^2^ = 1.6169, *p* = 0.2035; rs564481: χ^2^ = 0.1986, *p* = 0.6558).

A logistic regression analysis of the SNP variant rs1207568 at −395 G/A and rs564481 at 1818 C/T in the *Klotho* gene revealed no statistically significant differences in allele frequencies (*p* = 0.33, OR = 1.42 for *Klotho* rs1207568; *p* = 0.15, OR = 1.42 for *Klotho* rs564481), or genotype frequencies (*p* > 0.05, OR = 1.28–1.52 for *Klotho* rs1207568; *p* > 0.05, OR = 1.41–1.98 for *Klotho* rs564481) between the two studied groups. Confounding factors, such as age and sex, have no impact on *Klotho* rs1207568 and rs564481 genotype distributions and revealed no significant differences between the studied groups (Table 2).

Stratification of the glioma patients into subgroups according to grade have shown statistically significant differences in the *Klotho* rs1207568 (-395G/A) allele and genotype distributions. There was a statistically significantly increased frequency of the rs1207568A allele (*p* = 0.006, OR = 6.22), followed by frequency of the rs1207568 GA genotypes in the co-dominant model (*p* = 0.0064, OR = 6.13), dominant model (*p* = 0.0023, OR = 7.16) and over-dominant model (*p* = 0.011, OR = 5.41) in grade IV glioma patients when compared to grade II and III glioma patients (Table 3).

### 2.3. Association of Klotho rs1207568 and rs564481 Genotypes with Overall Survival in Glioma Patients

The association of *Klotho* rs1207568 and rs564481 polymorphisms with overall survival (OS) in patients with gliomas was also examined. The linear regression crude analysis, followed by analysis adjusted by sex, revealed no significant correlation of *Klotho* polymorphisms with OS in glioma patients in any genetic model (*p* > 0.05; Table 4).

### 2.4. Comparison of Serum Levels of sαKL in Glioma Patients and Healthy Controls

**Figure 1:**
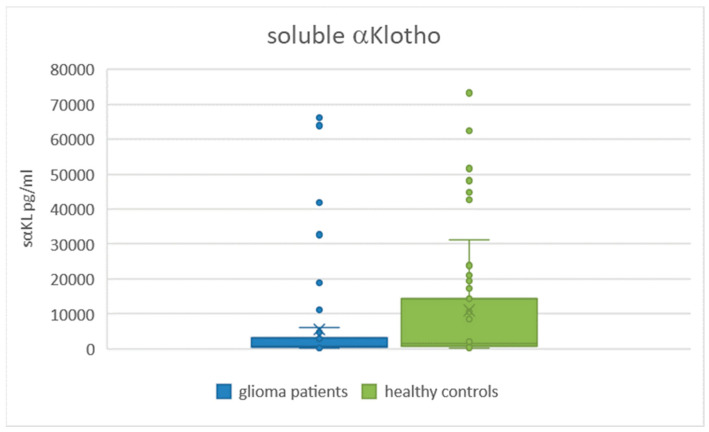
Serum levels of sαKL in glioma patients and healthy controls. *p* = 0.0036.

We analyzed the serum level of soluble alfa Klotho protein in a group of patients with gliomas and healthy controls. The serum levels of sαKL were significantly lower in the glioma patients (n = 55) (median (IQR): 672.16 (2583.88) pg/mL) than in the healthy controls (n = 47) (median (IQR): 1517.85 (13591.67) pg/mL, *p* = 0.0036) (Table 5, Figure 1).

We compared the serum levels of the sαKL in patient with gliomas at different stages of the disease. The level of sαKL did not significantly differ among the different gliomas grades (Table 6).

We also compared the serum levels of sαKL between gliomas of different grades and healthy controls. In grade II, the level of sαKL did not significantly differ in comparison with healthy controls (median; (IQR): 997.2 (4449.1) pg/mL vs. 1517.85; (13591.67) pg/mL, *p* = 0.21); however, we found statistically significantly lower serum levels in grade III (*p* = 0.034) and IV glioma patients (*p* = 0.0083) compared with the healthy controls (Table 7).

The serum level of sαKL in grade III (*p* = 0.034) and grade IV (*p* = 0.0083) glioma patients was statistically significantly lower than those in the healthy control individuals.

### 2.5. Correlation of Serum Level of sαKL with Overall Survival

The level of sαKL showed no statistically significant correlation with overall survival in the whole group of glioma patients, nor when comparing the level of sαKL in grade II and grade III + IV glioma patients (Table 8a–c).

**Figure 2:**
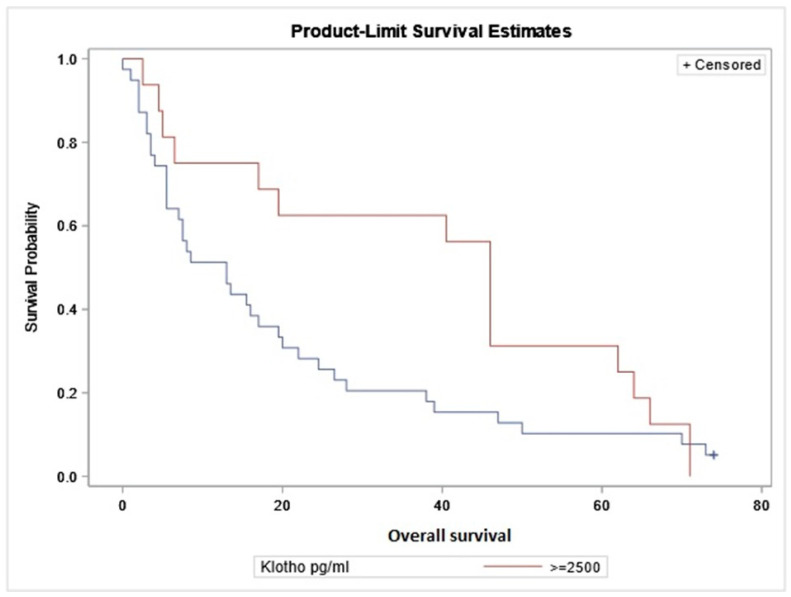
Kaplan–Meier survival curves of glioma patients according to sαKL. *x*-axis—survival time in months, *y*-axis—survival probability (test: Log rank *p* = 0.038). Blue line—overall survival in patients with Klotho levels below 2500 pg/mL.

We observed a significant difference in the overall survival of glioma patients when comparing patients with sαKL levels above and below the cut-off level of 2500 pg/mL. Patients with sαKL levels above 2500 pg/mL survived significantly longer than patients with sαKL below 2500 pg/mL (*p* = 0.038) (Figure 2).

The mean survival time (±SD) in patients (n = 39) with sαKL levels below 2500 pg/mL was 20.01 ± 21.99 months (95% CI: 12.88–27.15), while in patients with sαKL levels above 2500 pg/mL (n = 15), it was 40.73 ± 24.42 months (95% CI = 27.21–54.26; *p* = 0.01).

### 2.6. Correlation of Serum Levels of sαKL with the Levels of Candidate Biomarkers

We also made a correlation between the serum levels of sαKL and the levels of candidate biomarkers, as listed in Table 9.

In the group of glioma patients, seven biomolecules showed a positive correlation with the serum level of anti-aging sαKL (Table 10).

## 3. Discussion

The human *Klotho* gene located at chromosome 13q12 is composed of five exons and ranges over 50 kb. Early studies identified *Klotho* as an anti-aging gene [39]. Recent research on the functions of the *Klotho* gene has found its close correlation with malignant tumors. *Klotho* acts as an anti-oncogene in human lung cancer cell line A549 by inhibiting cell growth and promoting apoptosis [26].

Approximately 10 variations have been detected in the *Klotho* gene [40]. In our study, we analyzed two *Klotho* gene polymorphisms (*rs1207568* and *rs564481*) in glioma patients and healthy controls. A logistic regression analysis of the SNP variant rs1207568 at -395 G/A and rs564481 at 1818 C/T in the *Klotho* gene revealed no statistically significant differences in allele and/or genotype frequencies between the two studied groups. Confounding factors, such as age and sex, had no impact on the *Klotho* rs1207568 and rs564481 genotype distributions and revealed no significant differences between the studied groups. However, after stratification of patients into subgroups according to glioma grades, we found statistically significant differences in the *Klotho* rs1207568 (-395G/A) allele and genotype distributions. The frequency of allele A and genotype GA was significantly higher in the subgroup of grade IV in comparison with grade II + III glioma patients.

Our findings are similar with the results of Liu et al., who studied association of *Klotho* gene polymorphisms rs1207568 (-395G/A) and rs564481 (1818C/T) with colorectal cancer (CRC) development. They found that allele A and genotype GA of *Klotho* gene polymorphism rs1207568 (-395G/A) served as risk factors for CRC; however, C1818T polymorphism showed no effects on the pathogenesis of CRC [37]. Moreover, the study of Kamal et al. showed that the A allele of *Klotho rs1207568* is significantly associated with an increased risk of CRC development [38].

Klotho is mainly produced in the brain and kidneys. Many recent studies have proven that Klotho overexpression extends patient survival in different types of cancers [41,42,43,44,45]. On the other hand, we have very limited evidence about the role of Klotho in brain malignancies and in glial cells. Peshes-Yeloz et al. (2019) found that increased Klotho mRNA expression predicted longer survival of glioblastoma (GBM) patients. Klotho promoter hypermethylation was detected in 65% of the GBM samples and was significantly correlated with improved survival. In their work, the authors also demonstrated that GBM cell lines treated with soluble Klotho had significantly lower survival and proliferation rates compared with control cells [33].

Most published studies have described the loss of Klotho protein expression in human tumor tissues, but there is a relative paucity in the data relating to serum Klotho in solid tumors [46]. In addition, these studies had a small number of patients and were performed on a limited type of cancer, such as ESCC (esophageal squamous cell carcinoma), HCC (hepatocellular carcinoma), NSCLC (non-small cell lung cancer), RCC (renal cell carcinoma), DLBCL (diffuse large B-cell lymphoma) and multiple myeloma (MM) [47].

In our study, we found statistically significantly lower serum levels of sαKL in all the groups of glioma patients compared to the control group (*p* = 0.0036). We did not find any significant difference between grade II glioma patients and healthy controls; however, the serum levels of sαKL in grade III (*p* = 0.034) and grade IV glioma patients (*p* = 0.0083) were statistically significantly lower compared to healthy controls. According to these results, we may say that serum levels of sαKL in glioma patients may reflect the progression of the disease. We cannot compare our results with findings from other studies because, to our knowledge, the serum Klotho was not analyzed in glioma patients until now.

The Klotho serum levels were elevated in HCC and ESCC patients compared to healthy subjects; however, they were decreased in RCC patients [48,49,50]. This last finding is consistent with our results. In contrast to the analyses mentioned so far, a study undertaken by Pako et al. assessing plasma alpha-Klotho levels in 45 newly diagnosed lung cancer patients compared with 43 control subjects did not reveal any difference between these two groups [51]. Gigante et al., who analyzed preoperative blood serum from patients with RCC, found that reduced serum levels of alpha KL were statistically significantly associated with a higher tumor volume, Fuhrman grade and clinical stage [48].

MGMT (methylguanidine methyltransferase) promoter methylation reduces MGMT protein expression, which leads to increased sensitivity of tumor tissue to chemotherapy with temozolomide and thus improves patient survival [52]. However, we did not find any significant difference between the level of sαKlotho in patients with methylated and unmethylated MGMT promoter. In our previous study, we found lower levels of immunosuppressive molecule sHLA-G in glioma patients with methylated MGMT compared with patients with a non-methylated MGMT promoter [53]. In connection with these results, there are two valuable studies. Saaid et al. found that MGMT promoter methylation and gross total resection were key prognostic factors in their cohort of IDH (isocitrate dehydrogenase)-wildtype glioblastomas [54]. Similarly, a higher percentage of MGMT methylation was related to a lower preoperative tumor volume, larger extent of resection, increased progression free survival and overall survival in glioma patients [52].

Furthermore, we were interested in whether there is a correlation between the overall survival (OS) of patients and their serum αKL levels. Analyzing all of the groups of glioma patients, we did not find any statistically significant results. However, after dividing the patients into two subgroups according to the cut-off value of their sαKL = 2500 pg/mL, we found that the OS of patients with sαKL higher than 2500 pg/mL was significantly longer than the OS of patients with sαKL lower than 2500 pg/mL. Our results are consistent with studies analyzing renal cell carcinoma patients, where reduced serum levels of sαKL were associated with both decreased cancer-specific survival and progression-free survival [48].

Research on Klotho and cancer is usually conducted on animal models, followed by clinical trials involving only a limited number of patients. However, Qiao et al. (2022) realized a large-scale population-based analysis. Participants were selected from the NHANES (National Health and Nutrition Survey) in the years 2007–2016, excluding pregnant women, patients with chronic renal insufficiency and patients with incomplete data from a cancer questionnaire. The association of the serum Klotho level with cancer and mortality was analyzed. The authors found that serum Klotho had a tumor suppressor effect, and a higher level of Klotho concentration had a stronger effect. However, they did not observe any association between the serum Klotho level with all-cause mortality and cancer-specific mortality. The negative association of serum Klotho with pan-cancer varied significantly based on age, gender and BMI. The stratified analysis found that people aged 60–79, female, overweight and non-Hispanic whites or Mexican Americans were less likely to develop cancer [55].

In summary, the data indicate that we are just beginning to find out whether circulating Klotho could serve as a serum marker for the early diagnosis of various tumor types. The data confirming tumor regression in several “in vivo” models that are not Klotho-deficient support further evaluation of Klotho as a candidate therapeutic target [46].

We found only a limited number of studies analyzing the role of Klotho in brain tumors. According to our knowledge, most of the studies were devoted to research of Klotho protein expression and mRNA expression in tumor samples, but no work has investigated soluble αKlotho in serum. In our study, the role of sαKL in the sera of glioma patients was examined for the first time.

In our group of glioma patients, seven markers showed an interesting positive correlation with the serum level of sαKL, namely, angiogenic VEGF (*p* = 0.0008), CX3CL1 chemokine fractalkine (*p* = 0.0009), IFNγ (*p* = 0.003; main sustaining Th1 cytokine; role in anti-tumor immunity), GDNF (*p* = 0.0268), IL-6 (*p* = 0.0347), IL-4 (*p* = 0.0037) and IL-13 (*p* = 0.0004). To our knowledge, the correlations of αKL with the mentioned molecules have not been investigated in cancers until now.

## 4. Materials and Methods

### 4.1. Study Groups

This study involved 55 patients with gliomas (32 males, 23 females) older than 18 years (mean age of onset 54.38 ± 15.22 years) with partial or complete resection of a CNS tumor. Patients with an initial diagnosis and those experiencing a tumor relapse were analyzed. In our cohort, only patients with histologically confirmed glioma grades II, III and IV were included; all other histological types of tumors or other diagnoses were excluded. The diagnosis was validated by two neuropathologists based on the latest WHO classification criteria [56]. Patients were recruited from the Department of Neurosurgery, Faculty of Medicine, Comenius University and University Hospital Bratislava, Slovakia. The blood of glioma patients was obtained between the years 2015 and 2018. In this cohort, 15 patients had glioma grade II, 11 had grade III and 29 had grade IV. Blood samples were obtained from the patients on the morning of their surgical treatment day. The control group in our case-control study consisted of 140 unrelated volunteers (77 males and 63 females, with a mean age 41.93 ± 10.47 years). The control group for ELISA testing comprised 47 healthy subjects (mean age ± SD: 55.68 ± 10.83 years) without a significant difference of the mean age from the patient group. All the control subjects had no personal or family history of gliomas, other tumors or autoimmune or acute inflammatory diseases. They were recruited from a matched group within a larger population sample. All the patients and control subjects were Caucasians of Slovak descent. This study received approval from the Ethics Committee of the Faculty of Medicine, Comenius University in Bratislava, and University Hospital, Bratislava. All the investigations were conducted in accordance with the International Ethical Guidelines and the Declaration of Helsinki. Informed written consent for study enrollment and personal data management was obtained from all the patients and controls.

### 4.2. Genotyping

Genomic DNA was extracted from peripheral EDTA-treated blood samples (2 mL) using a modified salting-out method [57]. The *Klotho* rs1207568 (G or A allele at position -395) was analyzed using PCR, followed by restriction fragment length polymorphism (RFLP) analysis. Forward and reverse primer sequences, PCR conditions and the Hpy 188 III restriction enzyme (Thermo Fisher Scientific, Waltham, MA, USA) were used as reported by Liu et al. [37]. The PCR reaction revealed a 257 bp long product. After digestion, either an intact 257 bp PCR amplicon (allele G) or two fragments of 147 bp and 100 bp (allele A) were identified. The *Klotho* rs564481 (C or T allele at position 1818) was genotyped by PCR-RFLP as described by Liu et al. [37]. A 164 bp PCR fragment was amplified and subsequently digested with the restriction enzyme NsI (Thermo Fisher Scientific, Waltham, MA, USA). After cleavage, either an intact 164 bp PCR fragment with the C allele or two fragments of 136 bp and 28 bp with the T allele were produced.

### 4.3. Analysis of Serum/Plasma Levels of Investigated Biomarkers

The serum levels of soluble alfa Klotho protein and sTREM-1, plasma levels of VEGF, chemokine CX3CL1/fractalkine, GDNF, and pro-inflammatory cytokine IL-6, pro-inflammatory and Th1 cytokine IFN-γ, Th2 cytokines IL-4 and IL-13, and sHLA-G with immunosuppressive and anti-inflammatory activities were analyzed by a sandwich ELISA test (Human Elisa tests; all Fine Tests; Wuhan Fine Biotech Co., Ltd., Wuhan, China; except sHLA-G from EXBIO, Olomouc, Czech Republic) according to the manufacturer’s recommended procedure.

### 4.4. Statistical Analysis

The allele and genotype frequencies were assessed through direct counting using Excel software. The genotypes were tested for Hardy–Weinberg equilibrium using the chi-squared test. Statistical differences in allele and genotype frequencies between the two studied groups (patients with gliomas vs. control group) were assessed using the standard chi-squared test with InStat statistical software (GraphPad Software, version 3.10, San Diego, CA, USA). The *p* values, odds ratios (OR) and 95% confidence intervals (95% CI) were calculated for co-dominant, dominant, recessive and over-dominant inheritance models. The multivariate logistic regression analysis adjusted for sex and age as potential confounding variables was performed using the SNPstats web software available at https://www.snpstats.net/start.htm (accessed on 24 May 2024) [58]. To investigate the correlation between *Klotho* polymorphism and overall survival (OS), Student’s *t*-test with Welch correction was used. For statistical analysis of serum/plasma levels of selected molecules, programs InStat and BESH Stat were used. We used Student’s *t*-test, the Mann–Whitney test and the Spearman correlation to investigate the correlation of sαKlotho with the selected soluble molecules. A Kaplan–Meier analysis of αKlotho levels on OS in glioma patients and a log-rank test were performed, as well. A *p* value of <0.05 was set as statistically significant.

## 5. Conclusions

By analyzing genomic DNA isolated from peripheral blood samples, we found that stratifying glioma patients into subgroups according to glioma grades has shown statistically significant differences in *Klotho* rs1207568 (-395G/A) allele and genotype distributions. There was a significantly increased frequency of the rs1207568A allele, followed by frequency of the rs1207568 GA genotypes in a co-dominant model, dominant model and over-dominant model in grade IV glioma patients when compared to grade II and III glioma patients.

The serum levels of soluble α Klotho (sαKL) were significantly lower in glioma patients than in healthy controls, mainly in grade III and grade IV glioma patients. The OS of patients with a level of serum sαKL higher than 2500 pg/mL was significantly longer than the OS of patients with a level of serum sαKL lower than 2500 pg/mL. Moreover, seven biomarkers—VEGF, fractalkine, IFN-γ, GDNF, IL-6, IL-4 and IL-13—showed a significant positive correlation with the serum level of sαKL.

We are only at the beginning of our journey to fully understand the role of Klotho in gliomas and other brain tumors. At the same time, it would be highly beneficial for patients to determine if αKlotho could serve as a serum marker for early diagnosis, therapy monitoring or prognosis.

## Figures and Tables

**Table 1:** Characteristics of glioma patients.

**Patients**	**n**	**Mean Age ± SD**
All gliomas	55	54.38 ± 15.22
Sex (male/female)	32/23	49.63 ± 15.25/61.09 ± 12.55
Grades (male/female)		
G. II	11/4	40.33 ± 13.54
G. III	7/4	50.44 ± 10.14
G. IV	14/15	64.14 ± 9.59
Primary diagnosis	42	
Recidivism	9	
Not analyzed	4	
**Survival time**	**(months)**	
Overall	25.3 ± 24.36	
Grade II	50.47 ± 24.40	
Grade III	32.45 ± 20.14	
Grade IV	9.57 ± 9.28	

G—grade, n—number of patients, SD—standard deviation.

**Table 2:** *Klotho* rs1207568 and rs564481 allele and genotype frequencies in patients with gliomas and healthy control subjects.

SNP/ Model	Allele/ Genotype	Gliomas (n = 55)	Controls (n = 140)	Univariate Analysis		Multivariate Analysis	
				*p*	OR (95% CI)	*p*	OR (95% CI)
rs1207568	G	91 (82.73%)	244 (87.14%)				
-395G/A	A	19 (17.27%)	36 (12.86%)	0.33	1.42 (0.77–2.59)	-	-
	GG	38 (69.09%)	108 (77.14%)		1.00		1.00
Co-dominant	GA	15 (27.27%)	28 (20.00%)	0.51	1.52 (0.74–3.15)	0.80	1.33 (0.57–3.10)
	AA	2 (3.64%)	4 (2.86%)		1.42 (0.25–8.07		0.94 (0.12–7.60)
	GG	38 (69.09%)	108 (77.14%)		1.00		1.00
Dominant	GA + AA	17 (30.91%)	32 (22.86%)	0.25	1.51 (0.75–3.02)	0.55	1.28 (0.57–2.86)
	GG + GA	53 (96.36%)	136 (97.14%)		1.00		1.00
Recessive	AA	2 (3.64%)	4 (2.86%)	0.78	1.28 (0.23–7.21)	0.90	0.87 (0.11–7.07)
	GG + AA	40 (72.73%)	112 (80.00%)		1.00		1.00
Over-dominant	GA	15 (27.27%)	28 (20.00%)	0.28	1.50 (0.73–3.09)	0.50	1.34 (0.58–3.09)
HWE (χ^2^/*p*)		0.11/0.73	1.62/0.20				
rs564481	C	61 (55.45%)	179 (63.93%)				
1818C/T	T	49 (44.55%)	101 (36.07%)	0.15	1.42 (0.91–2.23)	-	-
	CC	15 (27.27%)	56 (40.00%)		1.00		1.00
Co-dominant	CT	31 (56.36%)	67 (47.86%)	0.23	1.73 (0.85–3.52)	0.16	2.05 (0.91–4.61)
	TT	9 (16.36%)	17 (12.14%)		1.98 (0.74–5.31)		2.19 (0.72–6.66)
	CC	15 (27.27%)	56 (40.00%)		1.00		1.00
Dominant	CT + TT	40 (72.73%)	84 (60.00%)	0.09	1.78 (0.90–3.52)	0.058	2.08 (0.96–4.52)
	CC + CT	46 (83.64%)	123 (87.86%)		1.00		1.00
Recessive	TT	9 (16.36%)	17 (12.14%)	0.44	1.42 (0.59–3.40)	0.49	1.42 (0.53–3.79)
	CC + TT	24 (43.63%)	73 (52.14%)		1.00		1.00
Over-dominant	CT	31 (56.36%)	67 (47.86%)	0.28	1.41 (0.75–2.64)	0.19	1.61 (0.79–3.29)
HWE (χ^2^/*p*)		1.09/0.29	0.19/0.66				

Allele and genotype frequencies are presented as absolute numbers with percentages in parentheses. OR—odds ratio, CI—confidence interval, n—number, HWE—Hardy–Weinberg equilibrium. Univariate analysis is based on χ^2^ test. Multivariate analysis is adjusted by sex and age. *p* < 0.05 was considered statistically significant.

**Table 3:** *Klotho* rs1207568 allele and genotype frequencies in patients with grade II and III vs. grade IV gliomas.

SNP/ Model	Allele/ Genotype	Grade IV (n = 29)	Grade II + III (n = 26)	Univariate Analysis		Multivariate Analysis	
				*p*	OR (95% CI)	*p*	OR (95% CI)
rs1207568	G	42 (72.41%)	49 (94.23%)				
-395G/A	A	16 (27.59%)	3 (5.77%)	**0.006**	6.22 (1.69–22.84)	-	-
	GG	15 (51.72%)	23 (88.46%)		1.00		1.00
Co-dominant	GA	12 (41.38%)	3 (11.54%)	**0.0064**	6.13 (1.48–25.44)	**0.029**	10.90 (1.39–85.53)
	AA	2 (6.90%)	0 (0.00%)		NA (0.00—NA)		NA (0.00—NA)
	GG	15 (51.72%)	23 (88.46%)		1.00		1.00
Dominant	GA + AA	14 (48.28%)	3 (11.54%)	**0.0023**	7.16 (1.75–29.20)	**0.0085**	11.59 (1.51–89.19)
	GG + GA	27 (93.10%)	26 (100.00%)		1.00		1.00
Recessive	AA	2 (6.90%)	0 (0.00%)	0.10	NA (0.00—NA)	0.43	NA (0.00—NA)
	GG + AA	17 (58.62%)	23 (88.46%)		1.00		1.00
Over-dominant	GA	12 (41.38%)	3 (11.54%)	**0.011**	5.41 (1.32–22.21)	**0.015**	9.99 (1.25–79.81)

Allele and genotype frequencies are presented as absolute numbers with percentages in parentheses. OR—odds ratio, CI—confidence interval, n—number, NA—not applicable. Univariate analysis is based on χ^2^ test. Multivariate analysis is adjusted by sex and age. *p* < 0.05 was considered statistically significant (typed bold).

**Table 4:** Association of *Klotho* rs1207568 and rs564481 genotypes with overall survival in glioma patients.

**Parameter** **rs1207568**	**G/G** **(n = 38)**	**G/A** **(n = 15)**	**A/A** **(n = 2)**	*p/p* * **CM**	*p/p* * **DM**	*p/p* * **RM**	*p/p* * **OD**
Overall survival, months ± SD	27.95 ± 25.80	18.53 ± 20.06	25.75 ± 28.64	0.46/0.31	0.23/0.12	0.98/0.65	0.21/0.16
**Parameter** **rs564481**	**C/C** **(n = 15)**	**C/T** **(n = 31)**	**T/T** **(n = 9)**	*p/p* * **CM**	*p/p* * **DM**	*p/p* * **RM**	*p/p* * **OD**
Overall survival, months ± SD	25.17 ± 26.06	25.21 ± 24.50	25.83 ± 23.78	1.00/0.93	0.98/0.70	0.94/0.89	0.98/0.81

CM—co-dominant model, DM—dominant model, OD—over-dominant model, RM—recessive model, SD—standard deviation. *p* < 0.05 was considered statistically significant., * *p*—*p* value adjusted for sex.

**Table 5:** The serum level of sαKL in glioma patients and healthy controls.

	Patients (n = 55)	Controls (n = 47)	*p*
**sαKL (pg/mL)** (median (IQR))	672.16 (2583.88)	1517.85 (13,591.67)	**0.0036**

sαKL—soluble alfa Klotho, IQR—interquartile range. *p* < 0.05 is statistically significant.

**Table 6:** Comparison of serum levels of sαKL in different grades of glioma.

	Grade II (n = 15) (Median (IQR))	Grade III (n = 11) (Median (IQR))	Grade IV (n = 29) (Median (IQR))	*p* (II vs. III)	*p* (II vs. IV)	*p* (III vs. IV)
**sαKL** (pg/mL)	997.2 (4449.1)	363.32 (2580.55)	598.1 (588.3)	0.24	0.47	0.43

sαKL—soluble alfa Klotho, IQR—interquartile range. *p* < 0.05 is statistically significant.

**Table 7:** The serum level of sαKL in grade III glioma patients and healthy controls.

	Grade II (n = 15)	Grade III (n = 11)	Grade IV (n = 29)	Controls (n = 47)	*p* (II vs. ctr)	*p* (III vs. ctr)	*p* (IV vs. ctr)
sαKL (pg/mL) (median (IQR))	997.2 (4449.1)	363.32 (2580.55)	598.1 (588.3)	1517.85 (13,591.67)	0.21	**0.034**	**0.0083**

sαKL—soluble alfa Klotho, IQR—interquartile range, ctr—control group. *p* < 0.05 is statistically significant.

**Table 8:** (**a**) Correlation of serum level of sαKL with overall survival of all glioma patients. (**b**) Correlation of serum level of sαKL with overall survival of grade II glioma patients. (**c**) Correlation of serum level of sαKL with overall survival of grade III + IV glioma patients.

**(a)**				
		**Spearman r**	**95% CI**	***p***
**sαKL** (pg/mL)	672.16 (2583.88)	0.195	−0.082–0.444	0.15
**Survival time** (months)	25.3 ± 24.36			
**(b)**				
		**Spearman r**	**95% CI**	***p***
**sαKL** (pg/mL)	1238.44 (4741.26)	0.211	−0.352–0.662	0.45
**Survival time** (months)	64 (32.5)			
**(c)**				
		**Spearman r**	**95% CI**	***p***
**sαKL** (pg/mL)	589.75 (941.36)	−0.067	−0.38–0.258	0.68
**Survival time** (months)	8.5 (16.5)			

Legend: sαKL—soluble alfa Klotho, CI—confidence interval. *p* < 0.05 is statistically significant.

**Table 9:** Serum/plasma levels of diagnostic molecules selected for profiling in glioma patients.

Molecules	Median (IQR)
**VEGF** (pg/mL)	92.76 (409.1)
**Fractalkine** (pg/mL)	582 (529.75)
**sTREM-1** (pg/mL)	33.2 (37.16)
**IFN-γ** (pg/mL)	18.29 (112.12)
**sHLA-G** (U/mL)	27.69 (39.95)
**GDNF** (pg/mL)	46.27 (340.18)
**IL-6** (pg/mL)	3 (9.37)
**IL-4** (pg/mL)	216.6 (298.4)
**IL-13** (pg/mL)	31.46 (175.64)

IQR—interquartile range, VEGF—vascular endothelial growth factor, IFN-γ—interferon gamma, HLA-G—human leukocyte antigen G, GDNF—glial-derived neurotrophic factor, IL—interleukin.

**Table 10:** Correlation of serum levels of sαKL with candidate biomarkers selected for serum/plasma profiling in glioma patients.

Correlation of	Spearman r	95% CI	*p*
sαKL with VEGF	0.475	0.215–0.673	**0.0008**
sαKL with fractalkine	0.431	0.188–0.625	**0.0009**
sαKL with sTREM-1	−0.0247	−0.2955–0.249	0.858
sαKL with IFN-γ	0.408	0.141–0.619	**0.003**
sαKL with sHLA-G	0.0436	−0.249–0.329	0.766
sαKL with GDNF	0.338	0.032–0.585	**0.0268**
sαKL with IL-6	0.302	0.015–0.543	**0.0347**
sαKL with IL-4	0.424	0.140–0.644	**0.0037**
sαKL with IL-13	0.509	0.245–0.703	**0.0004**

sαKL—soluble alfa Klotho, VEGF—vascular endothelial growth factor, IFN-γ—interferon gamma, HLA-G—human leukocyte antigen G, GDNF—glial-derived neurotrophic factor, TREM-1—triggering receptor expressed on myelocytes, IL—interleukin, CI—confidence interval. *p* < 0.05 is statistically significant.

## Data Availability

The data that supports the findings of this study are available within the article. The raw data of this study are available on request from the corresponding author due to privacy restrictions.
